# Methadone does not potentiate the effect of doxorubicin in canine tumour cell lines

**DOI:** 10.1002/vms3.266

**Published:** 2020-04-19

**Authors:** Claudia Cueni, Katarzyna J. Nytko, Pauline Thumser‐Henner, Mathias S. Weyland, Carla Rohrer Bley

**Affiliations:** ^1^ Division of Radiation Oncology Vetsuisse Faculty University of Zurich Zurich Switzerland; ^2^ Center for Clinical Studies at the Vetsuisse Faculty of the University of Zurich Zurich, Switzerland; ^3^ ZHAW School of Engineering Zurich University of Applied Sciences Winterthur Switzerland

**Keywords:** µ‐receptor, buprenorphine, cancer, dogs, flow cytometry, opioid receptor

## Abstract

Opioid receptor activation was shown to enhance the efficacy of anti‐neoplastic drugs in several human cancer cell lines. In these cell lines, doxorubicin increased the number of opioid receptors and methadone concurrently enhanced cellular doxorubicin uptake. Triggered through lay press and media, animal owners started to challenge veterinary oncologists with questions about methadone use in anti‐cancer therapy. Especially in veterinary medicine, where side effects of chemotherapy are tolerated to a lesser extent and hence smaller doses are given, agents potentiating chemotherapeutic agents would be an optimal approach to treatment. Canine transitional cell carcinoma cells (TCC, K9TCC), canine osteosarcoma cells (OSA, Abrams) and canine hemangiosarcoma cells (HSA, DAL‐4) were incubated with different combinations of methadone, buprenorphine and doxorubicin, in order to test inhibition of cell proliferation. Opioid receptor density was assessed with fluorescence‐activated cell sorting in drug native and doxorubicin pretreated cells. In TCC and OSA cell lines opioid receptor density increased after doxorubicin pretreatment. In combination treatment, however, we did not find significant potentiation of doxorubicin's inhibitory effect on proliferation in these cell lines. Neither was there a significant increase of the effect of doxorubicin when the opioids were added 24 hr before doxorubicin. Hence, we could not confirm the hypothesis that opioids increase the anti‐proliferative effect of the anti‐neoplastic drug doxorubicin in any of these canine tumour cell lines. The lack of effect on a cellular level does not warrant a clinical approach to use opioids together with doxorubicin in dogs with cancer.

## Graphical abstract

Triggered through lay press and media, animal owners started to challenge veterinary oncologists with questions about methadone use in anti‐cancer therapy. However, we could not confirm the hypothesis that opioids increase the anti‐proliferative effect of the anti‐neoplastic drug doxorubicin in any of these canine tumor cell lines. The lack of effect on a cellular level does not warrant a clinical approach to use opioids together with doxorubicin in dogs with cancer.

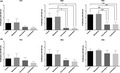

## 1 INTRODUCTION

Oncologists continue to search for possibilities to enhance the effectiveness of chemotherapeutic agents without adding toxicity to the patient. Recently, the opioid methadone was described to potentiate the pro‐apoptotic effect of doxorubicin, a commonly used anti‐neoplastic agent in human leukaemia and glioblastoma cells (HL‐60, CEM, Tanoue, Reh, Nalm6, A172) in vitro Friesen, Bacher, Hormann, Roscher, and Miltner (2011); Friesen, Hormann, & Roscher, 2014; Friesen, Roscher, & Hormann, 2013; Singh, Jayanthan, & Farran, 2011) Furthermore, the researchers showed also tumour‐growth inhibitory properties in co‐treated patient‐derived acute lymphoblastic leukemia (ALL‐SCID6) xenografts in vivo (mouse model) (Friesen et al., 2013). Two distinct mechanisms were proposed as chemo‐enhancing properties: First, methadone increases the intracellular content and hence activity of doxorubicin even in drug‐resistant tumour cells, secondly, doxorubicin increases opioid receptor expression in leukaemia cells (Friesen et al., 2013).

When these findings were spread publicly through lay press and media, cancer patients started to confront physicians and pharmacists, willing to use methadone in their cancer treatment (Theile & Mikus, 2018). Equally, animal owners started to challenge veterinary oncologists with questions about methadone use in anti‐cancer therapy. Some veterinarians even started to use the combination of methadone with chemotherapy in cancer bearing dogs on clients’ request.

In animals or cancer cell lines of pet animals, the interaction of opioids with anti‐neoplastic agents has not been investigated to date. In this study, we wanted to explore if opioid receptors can be found on canine tumour cells and whether an interaction of opioids and doxorubicin can be observed. Methadone is a synthetic opioid agonist and binds to the opioid μ‐receptor. The drug is regularly used in veterinary medicine, mostly for perioperative analgesia and to relieve short‐ to intermediate‐term painful conditions (Grimm, Lamont, Tanquilli, Greene, & Robertson, 2015; Ingvast‐Larsson, Holgersson, Bondesson, Lagerstedt, & Olsson, 2010). However, opioids in general underlie strict regulatory guidelines and their long‐term use in an outpatient setting is not as common as in man.

Doxorubicin, an anthracycline antibiotic, is a frequently administered anti‐neoplastic agent in veterinary medicine (Arcamone, Cassinelli, & Fantini, 1969; Arcamone, Cassinelli, & Franceschi, 1972; Withrow, Vail, & Page, 2013). Applied in various species, the dose of doxorubicin is limited to dosages of ≤30 mg/m^2^ due to mostly gastrointestinal and haematologic toxicity. Furthermore, a cumulative dose of 120–150 mg/m^2^ can result in specific cardiac toxicity in dogs (Ogilvie, Richardson, & Curtis, 1989; Sparano, Gordon, Hall, Iatropoulos, & Noble, 1982; Vanvleet & Ferrans, 1980). While initially effective in many disease entities, over time, drug resistance will often occur (Shahi et al., 2015; Zandvliet, Teske, Schrickx, & Mol, 2015; Zandvliet, Teske, & Schrickx, 2014). To overcome or reverse the resistance towards the drug or to enhance the efficacy of doxorubicin without concurrently enhancing clinical side effects would be of great interest in veterinary medicine.

Under our first hypothesis, i.e. that canine tumour cells from various diseases express opioid receptors, we tested several canine cell lines (transitional cell carcinoma (TCC), osteosarcoma (OSA) and hemangiosarcoma (HSA)) by flow cytometric analysis before and after doxorubicin exposure. Next, we tested if the concurrent use of opioids in canine tumour cell lines in vitro enhances effects of doxorubicin, measured with proliferation assay. The resulting data will be used for future decisions on whether opioids should be investigated deeper with an aim of a possible use in a clinical setting in dog cancer patients with specific neoplastic conditions receiving doxorubicin.

## 2 MATERIALS AND METHODS

First, presence and expression of opioid receptors on canine transitional cell carcinoma, canine osteosarcoma and canine cells hemangiosarcoma before and after the treatment with doxorubicin was investigated through flow cytometry. Then, different dosages of doxorubicin, methadone and buprenorphine were tested on all three cell lines. Afterwards, the selected doses were used for different drug combinations, which were examined by means of a cell proliferation assay.

### 2.1 Drugs and reagents

Methadone hydrochloride (Methadon Streuli^®^) was obtained from Streuli Pharma AG (Uznach, Switzerland) and buprenorphine hydrochloride (Temgesic^®^) was obtained from Indivior Schweiz AG (Baar, Switzerland). Both are aqueous solutions in disposable ampoules. For each experiment a new ampoule was opened.

Doxorubicin hydrochloride (Adriblastin^®^ RD/‐Solution) was obtained from Pfizer AG (Zürich, Switzerland). Each vial was used three to six times and stored at 4°C for a maximum of 3 weeks. Adriblastin is an aqueous solution.

Naloxone fluorescein acetate was obtained from Tocris, Bio‐Techne (Zug, Switzerland) and was dissolved in dimethylsulfoxide.

### 2.2 Cell lines

Canine transitional cell carcinoma cells (K9TCC) was obtained from Prof. Knapp, Purdue (PU, Indiana, USA), canine osteosarcoma cell (Abrams) were obtained from Prof. Rebhurn, (UCD, California, USA) and canine hemangiosarcoma cells (DAL‐4) were obtained from Kerafast Inc. (Massachusetts, USA). The K9TCC and Abrams cells were grown in Dulbecco's modified eagle's medium (Gibco^™^) containing 10% fetal bovine serum (FBS) (Gibco^™^), 100 units/ml of penicillin (Gibco^™^), 100 μg/ml of streptomycin (Gibco^™^) and 10 mM of 4‐(2‐hydroxyethyl)‐1‐piperazineethanesulfonic acid buffer (Gibco^™^) and incubated at 5% CO_2_ and 37°C. The three cell lines are described as TCC (transitional cell carcinoma, K9TCC), OSA (osteosarcoma, Abrams) and hemangiosarcoma (HSA, DAL‐4) in figures and text.

The DAL‐4 cells were grown in Ham F‐12 (Gibco^™^) containing 10% FBS (Gibco^™^), 100 μg/ml of primocin, 0.05 mg/ml of endothelial cell growth supplement, 0.1 mg/ml of heparin (Sigma‐Aldrich^®^), 10 mM of HEPES buffer (Gibco^™^) and incubated at 37°C in 5% CO_2_ humidified incubator. All cell lines were free of mycoplasma.

### 2.3 Detection of opioid receptors

Cells were incubated for 72 hr with either doxorubicin or saline. The concentration of doxorubicin was 0.5 μg/ml for TCC, 0.15 μg/ml for OSA and 0.05 μg/ml for HSA. The cells were trypsinized, centrifuged and resuspended in PBS/1% FBS containing naloxone fluorescein acetate (0.05nM). The cells were incubated for 30 min at RT in darkness. After incubation the cells were washed, centrifuged and resuspended in cold PBS/1% FBS. CytoFLEX S flow cytometer (Institute of Virology, Vetsuisse Faculty of the University of Zurich, Switzerland) was used for the flow cytometer analysis. The flow cytometry results were analysed by FlowJo 10.6.1 software.

### 2.4 Proliferation assay

The proliferation assay was performed with the cell counting kit CCK‐8 according to the manufacturer's protocol (Dojindo Laboratories, Kumamoto, Japan). CCK‐8 reagent is a ready for use solution, which allows determination of the number of viable cells in proliferation assays. Five hundred cells for OSA and TCC cells, and 2000 cells for HSA cells per well were seeded into 96‐well plates. For initial dose finding, cells were treated with 10, 5 and 1 μg/ml of methadone, 0.2, 0.5, 1 μg/ml of buprenorphine and 0.5, 0.1, 0.05, 0.015, 0.01, 0.005 μg/ml of doxorubicin. The cells were incubated up to 72 hr and the proliferation was measured at time points of 0, 24, 48 and 72 hr. At each time point, 10 μl of the CCK‐8 solution was given to the corresponding wells and the absorbance was measured 4 hr later at 450 and 600 nm using microplate reader Epoche 2 (BioTek).

For the combination studies, methadone or doxorubicin was added to cells for 24 hr prior to adding the combination compound doxorubicin and methadone, respectively. The concentrations and order of the compound addition for each cell line are indicated in the figure legend. The cell proliferation was measured 72 hr after adding the first compound.

### 2.5 Statistical analysis

**Figure 1:**
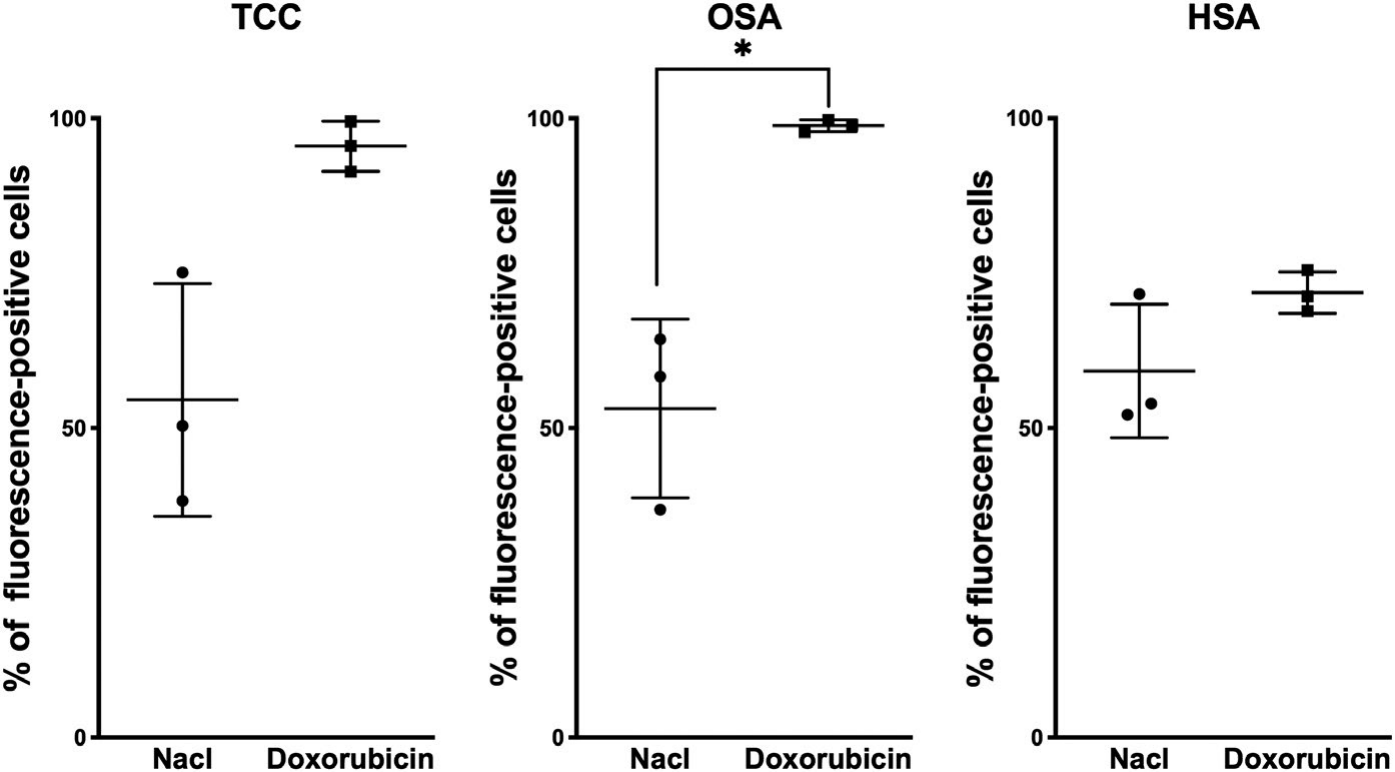
Effect of pretreatment with Doxorubicin on opioid receptor expression in canine transitional cell carcinoma (TCC), canine osteosarcoma Abrams (OSA) and canine hemangiosarcoma DAL‐4 (HSA). Mean ± *SD* of three experiments performed independently is shown

**Figure 2:**
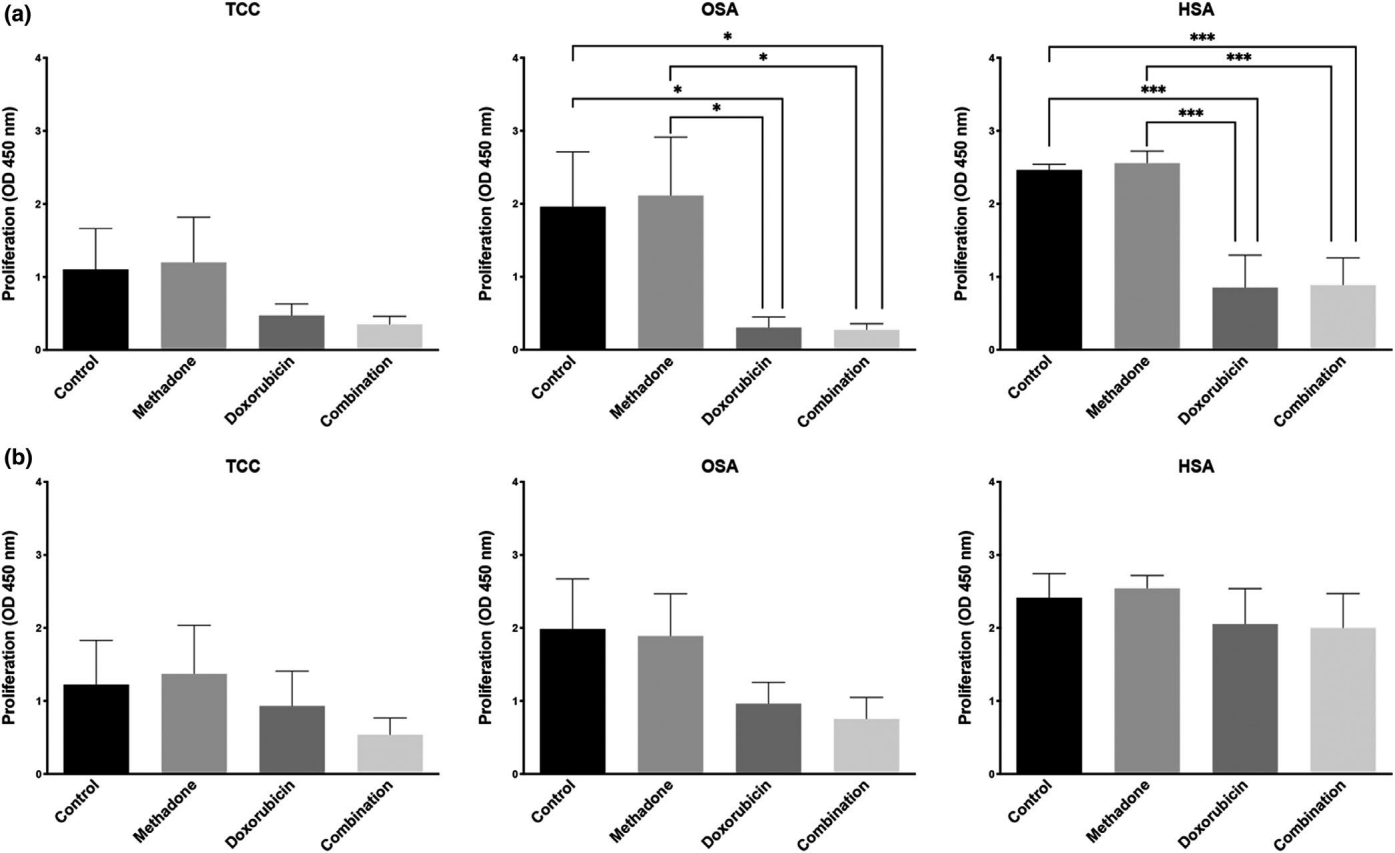
Combination of methadone and doxorubicin does not result in enhanced cancer cell growth inhibition in canine transitional cell carcinoma (TCC), canine osteosarcoma Abrams (OSA) and canine hemangiosarcoma DAL‐4 (HSA). Cells pretreated with doxorubicin first (a) and methadone first (b) were incubated for 72 hr and cell viability was measured. Mean ± *SD* of three experiments performed independently is shown

Statistical analyses were performed using R (The R Foundation for Statistical Computing, version 3.6.0, 2019) along with the multcomp package (version 1.4–10) for post‐hoc tests. For the detection of opioid receptors by fluorescence‐activated cell sorting, two‐tailed unpaired Student *t*‐tests with the Welch–Satterthwaite approximation were used (see Figure 1). The analysis of the data obtained from proliferation assays was done with one‐way ANOVA. In the combination study, Tukey's range test was used for pairwise comparison of the four factor levels (see Figure 2 and Figure S3). In the dose finding study, the three respective doses were compared to the control using Dunnett's test at each time point and for each compound (see Figure S2). *p* values below .05 were considered statistically significant and denoted with a star (*). Two stars (**) were used for p values below 0.01 and three stars (***) were used for p values falling below 0.001.

## 3 RESULTS

### 3.1 Expression of opioid receptor in canine cancer cell lines

First, we investigated the presence and expression of opioid receptors on canine transitional cell carcinoma, canine osteosarcoma and canine hemangiosarcoma cells through flow cytometry. Flow cytometry revealed receptors on all three tested cell lines. In all tested cell lines, 35%–80% of the cells expressed opioid receptors in untreated cells (Figure S1). After incubation with doxorubicin for 72 hr TCC and OSA increased opioid receptor expression almost twofold, to over 90%, which was significantly higher than in the control group (Figure 1). HSA showed no significant enhancement of the receptor density after doxorubicin treatment (Figure 1).

### 3.2 Effects of different opioid and doxorubicin concentrations on canine cancer cell line proliferation

We tested anti‐proliferative effects of increasing doses of methadone or buprenorphine and doxorubicin on the three cell lines in order to determine the optimal concentration for the combinatorial experiments. Methadone and buprenorphine did not inhibit cell proliferation of all cell lines tested up to concentration of 10 μg/ml and 1 μg/ml, respectively (Figure S2a and b). Doxorubicin had strong, concentration dependent, inhibitory effect on cell proliferation in all three cell lines tested (Figure S2c). The cell lines, however, were unequally sensitive towards doxorubicin: the approximately 50% inhibitory concentration at 48 hr was highest in TCC with 0.500 μg/ml, fivefold lower in OSA (0.100 μg/ml) and 33.3‐fold lower in HSA cell lines (0.015 μg/ml). We then chose the dose of each compound for the combinatorial experiments (Table 1 and 2). The chosen concentrations should only minimally inhibit cell proliferation alone, in order to be able to observe the effects of combinations. Furthermore, the applied dose had to fulfill the criterion to potentially produce clinically achievable plasma levels in dogs. Based on our findings (Figure S2a and b), we selected the same opioid dose for all cell lines to be used in combination experiments.

**Table 1:** Doses of methadone and doxorubicin for experiments

	Methadone	Doxorubicin
Transitional cell carcinoma (K9TCC)	3 μg/ml	0.500 μg/ml
Osteosarcoma (Abrams)	3 μg/ml	0.100 μg/ml
Hemangiosarcoma (DAL‐4)	3 μg/ml	0.015 μg/ml

**Table 2:** Doses of buprenorphine and doxorubicin for experiments

	Buprenorphine	Doxorubicin
Transitional cell carcinoma (K9TCC)	0.5 μg/ml	0.500 μg/ml
Osteosarcoma (Abrams)	0.5 μg/ml	0.100 μg/ml
Hemangiosarcoma (DAL‐4)	0.5 μg/ml	0.015 μg/ml

### 3.3 Drug combinations show no potentiation of anti‐proliferative effect

When the cells were pretreated with doxorubicin and methadone was added 24 hr later, we did not find a potentiation of doxorubicin's inhibitory effect on proliferation in TCC, OSA or HSA cell lines (Figure 2a). Pretreatment with methadone for 24 hr did not enhance the cytotoxic effect of doxorubicin either (Figure 2b). Similar to methadone, combination of doxorubicin and buprenorphine did not result in enhanced anti‐proliferative effect in any of the combinations tested, irrespective if doxorubicin or buprenorphine was added first (Figure S3a and b).

## 4 DISCUSSION

The opioid methadone recently gained much attention as an anti‐neoplastic compound, possibly potentiating in vitro and in vivo efficacy of doxorubicin (Friesen, Roscher, Alt, & Miltner, 2008; Friesen et al., 2013; Singh et al., 2011). The effects, however, were not constantly shown. From a pharmacological point of view, the in vitro anti‐neoplastic effects mostly occurred at unrealistic clinical dose levels and the mode of action in terms of receptors and pathways is unclear (Brawanski et al., 2018; Theile & Mikus, 2018). In addition, no randomized, controlled and well‐powered clinical studies on a possible anti‐neoplastic efficacy of methadone are available. The few retrospective investigations show no impact on progression‐free survival or overall survival on cancers with glioblastoma multiforme (Onken, Friesen, Vajkoczy, & Misch, 2017) or other cancers (Reddy, Schuler, & Cruz, 2017). It is of note that for human cancer patients, methadone is neither approved nor recommended as an anti‐cancer treatment. Various specialist associations reject the use of methadone as an antitumour treatment from a medical and ethical point of view (Kreye, Masel, Hackner, Stich, & Nauck, 2018).

In this study the number of opioid receptors was moderate and increased after doxorubicin exposure in the canine TCC and OSA, but not in the HSA cell line. Cell lines with high opioid receptor density such as human leukaemia cells were found to respond stronger to methadone or combination treatment than cells with only moderate opioid receptor expression (Friesen et al., 2008, 2013). The receptor density could be cell line or even patient‐specific. In terms of direct growth inhibition by opioids, some established tumour cell lines showed increased apoptosis at low methadone concentrations of 1 μg/ml, together with temozolomide. In others, cell lines derived from human glioblastoma multiforme‐patient samples, had no significant quantitative differences in the μ‐opioid receptor expression, and only a decrease of cell viability in a higher dose‐dependent range (15–45 μg/ml) was found (Brawanski et al., 2018). In another cell culture model also high doses of methadone (10–30 μM, about 3‐–μg/ml) were needed to reduce glioblastoma cell viability (Oppermann, Matusova, & Glasow, 2019). Various doses of methadone did not affect cell viability of melanoma cell lines grouped into μ‐opioid receptor density (OPRM1 high, medium, negative). The concurrent cisplatin and methadone treatment resulted in a slightly decreased cell viability of 10%–20% in the high receptor‐dense cells. The effect, however, could not be reproduced with temozolomide as anti‐neoplastic agent (Bruggen, Mangana, & Irmisch, 2018).

We could, however, not confirm the hypothesis that opioids increase the potency of doxorubicin in any of the three investigated cancer cell lines of dogs. Comparably, no anti‐neoplastic effect was observed in several established and primary human glioblastoma cell lines using combinations of methadone with temozolomide (Brawanski et al., 2018), irradiation or both (Oppermann et al., 2019). A reasonable concentration of methadone in cell line conditions, which can be reached using clinically tolerable dosage in man is around 0.3–1.3 μg/ml (Brawanski et al., 2018; Inturrisi, Colburn, Kaiko, Houde, & Foley, 1987). For our experiments, we chose the dose of methadone (3 μg/ml) and buprenorphine (0.5 μg/ml) in slightly higher range (Abbo, Ko, & Maxwell, 2008; Ingvast‐Larsson et al., 2010), and verified that the chosen dose did not inhibit tumour cell proliferation. A direct inhibition of cell proliferation by methadone with increasing doses is possible, but was only observed at doses higher than 3 μg/ml, around 10 μg/ml (Brawanski et al., 2018; Friesen et al., 2013; Oppermann et al., 2019).

We selected the herein used three dog cell lines of low, moderate and high sensitivity to doxorubicin treatment. As clinical diseases in dogs, hemangiosarcoma and osteosarcoma are treated with doxorubicin as a single agent or in combination with other anti‐neoplastic drugs (Kent, Strom, London, & Seguin, 2004; Mauldin, Matus, Withrow, & Patnaik, 1988; Ogilvie, Powers, Mallinckrodt, & Withrow, 1996; Sorenmo, Jeglum, & Helfand, 1993). The former cell line shows high sensitivity and the latter moderate sensitivity (fivefold less than hemangiosarcoma) towards growth inhibition with doxorubicin. Transitional cell carcinomas in the dog are usually not treated with doxorubicin in a clinical setting and showed lowest sensitivity as a cell line (33‐fold less than hemangiosarcoma) (Arnold, Childress, & Fourez, 2011; Marconato et al., 2011; Shapiro, Kitchell, Fossum, Couto, & Theilen, 1988). Inhibition of individual cell line's proliferation by doxorubicin was measured 48 hr after treatment start to reveal concentration that inhibits the cells by 50%–70%.

Buprenorphine was used as a second opioid to test. The oral bioavailability of methadone in dogs is described to be below detection, owing to the first pass effect (Kukanich, Kukanich, & Rodriguez, 2011; Kukanich, Lascelles, Aman, Mealey, & Papich, 2005). Buprenorphine applied transmucosally, on the contrary, has a bioavailability between 38% and 47%, which could be useful and convenient for an outpatient setting. Because opioids as methadone and buprenorphine are bound to the strict federal law of narcotics, an outpatient trial would only be possible if the opioids can be given orally or transmucosally.

The cell lines used in this study express moderate amounts of opioid receptors, and its expression increased to over 90% after 72 hr of incubation with doxorubicin in the transitional carcinoma and the osteosarcoma cell line. Nevertheless, no positive or negative effect on cell proliferation could be observed after co‐treatment with opioids. Friesen et al. (2011, 2013, 2014) suspected the effect of methadone to be achieved by the μ‐receptor activation on tumour cells, with an unknown mechanism. The expression levels of the classical opioid receptor were assessed through an indirect approach with the fluorescein‐labeled naloxone. This is a limitation in our approach, as we neither provide a direct, for example antibody‐based evaluation of μ‐opioid receptor, nor provide evidence of functionality of μ‐receptors with overexpression or knock‐down. Naloxone is a non‐selective opioid antagonist and could have bound any opioid receptor in flow cytometry. In the worst case, the specific absence of the μ‐receptor could even have been a possible reason why in our study opioids and doxorubicin combination treatment showed no benefit. Furthermore, other receptors could be involved in sensitization or induction of apoptosis by opioids (Li, Li, & Zhang, 2010; Yin, Mufson, Wang, & Shi, 1999).

However, along with the findings of our colleagues from human medicine (Brawanski et al., 2018; Bruggen et al., 2018; Inturrisi et al., 1987; Kreye et al., 2018; Oppermann et al., 2019; Theile & Mikus, 2018) we cannot advocate the clinical use of opioids to enhance doxorubicin's efficacy in dogs with tumours. While we found an increase in opioid receptors in TCC and OSA, none of the combination treatments indicated additional inhibition of cellular proliferation. Furthermore, also in dogs, opioids and specifically methadone as a long‐term treatment can have considerable side effects such as inappetence, nausea, constipation (Frey & Löscher, 2010). Additional side effects could have a massive impact on quality of life of dog patients undergoing chemotherapy.

## 5 CONCLUSIONS

The lack of effect on a cellular level does not warrant a clinical approach to use opioids together with doxorubicin in dogs with cancer. In case of further pursuit of such combined approach it should be proactively considered to use an orally bioavailable variant such as buprenorphine.

## AUTHOR CONTRIBUTION

**Claudia Cueni:** Conceptualization; Formal analysis; Investigation; Writing‐original draft. **Katarzyna J Nytko:** Formal analysis; Investigation; Supervision; Writing‐review & editing. **Pauline Thumser‐Henner:** Investigation; Writing‐review & editing. **Mathias S. Weyland:** Formal analysis. **Carla Rohrer Bley:** Conceptualization; Project administration; Supervision; Writing‐original draft; Writing‐review & editing. 

## Supporting information

Fig S1

Fig S2

Fig S3

Fig S4

## Data Availability

The data that support the findings of this study are available on request from the corresponding author.
