# Supplementary figures and images for: Methadone does not potentiate the effect of doxorubicin in canine tumour cell lines
Source: Vet Med Sci. 2020 Apr 19;6(3):283–9. doi: 10.1002/vms3.266 (PMC7397897; doi:10.1002/vms3.266)

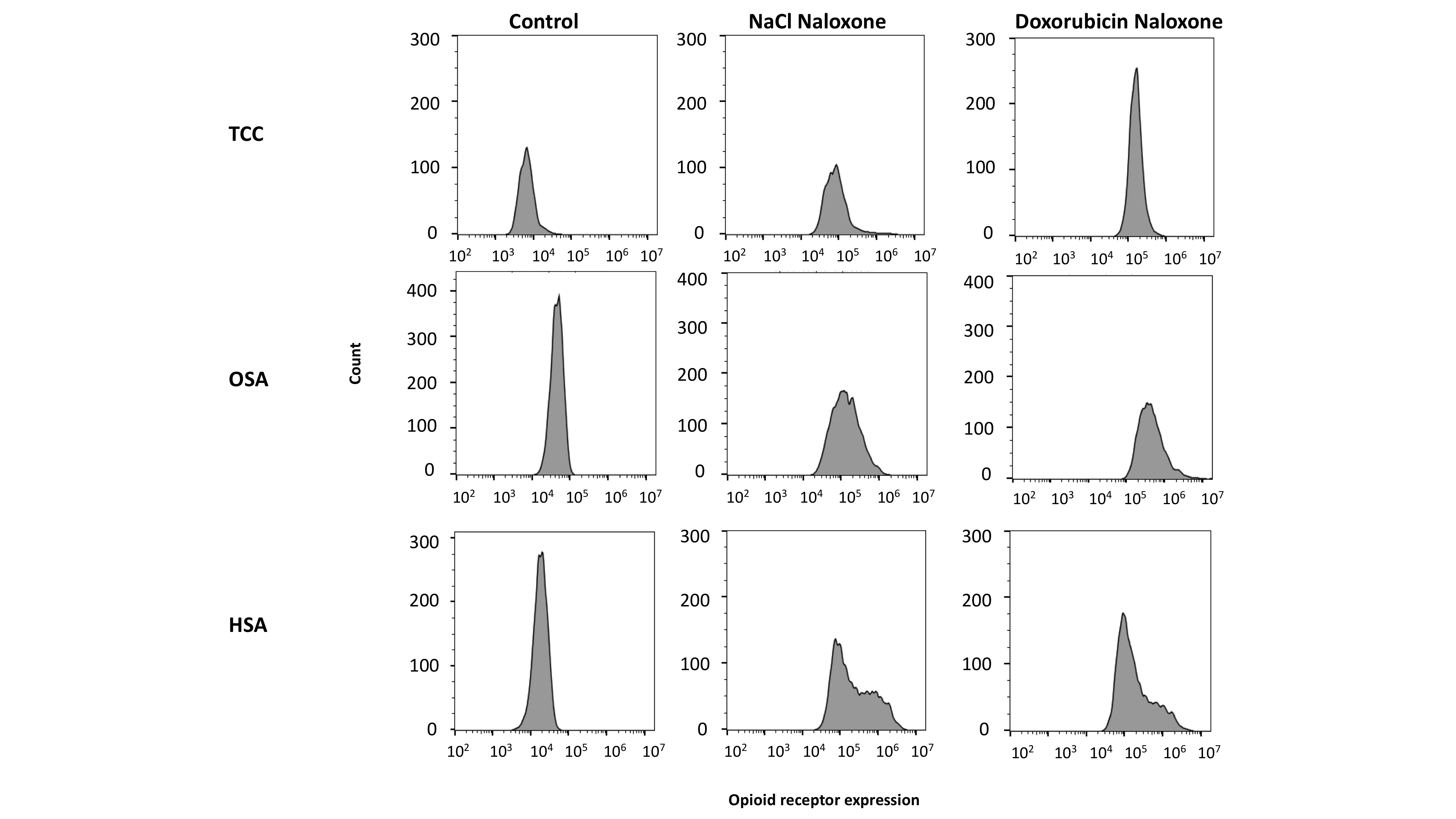

Supplement: Supplementary file 1 — Fig S1 [file VMS3-6-283-s001.tiff]

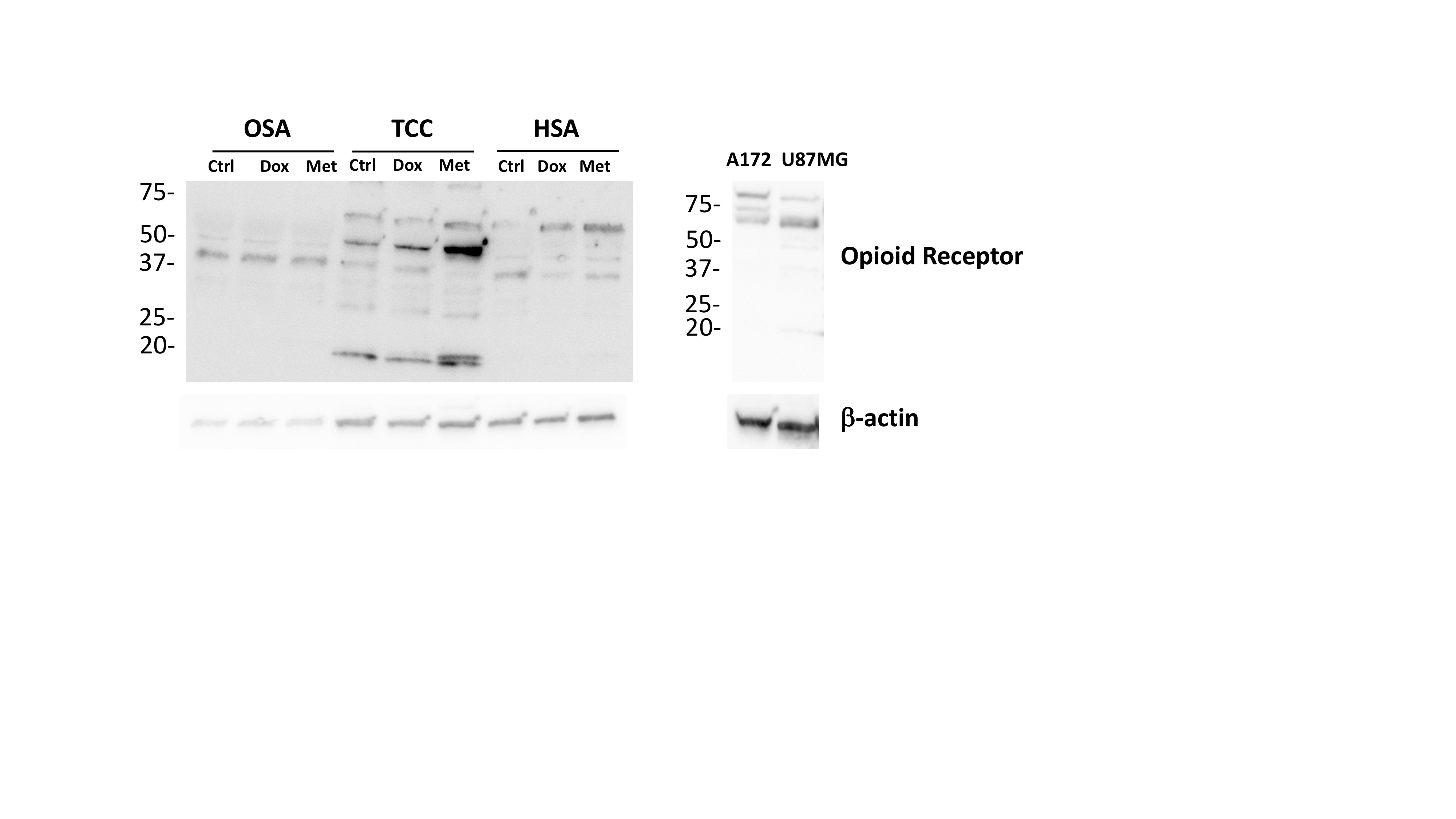

Supplement: Supplementary file 2 — Fig S2 [file VMS3-6-283-s002.tiff]

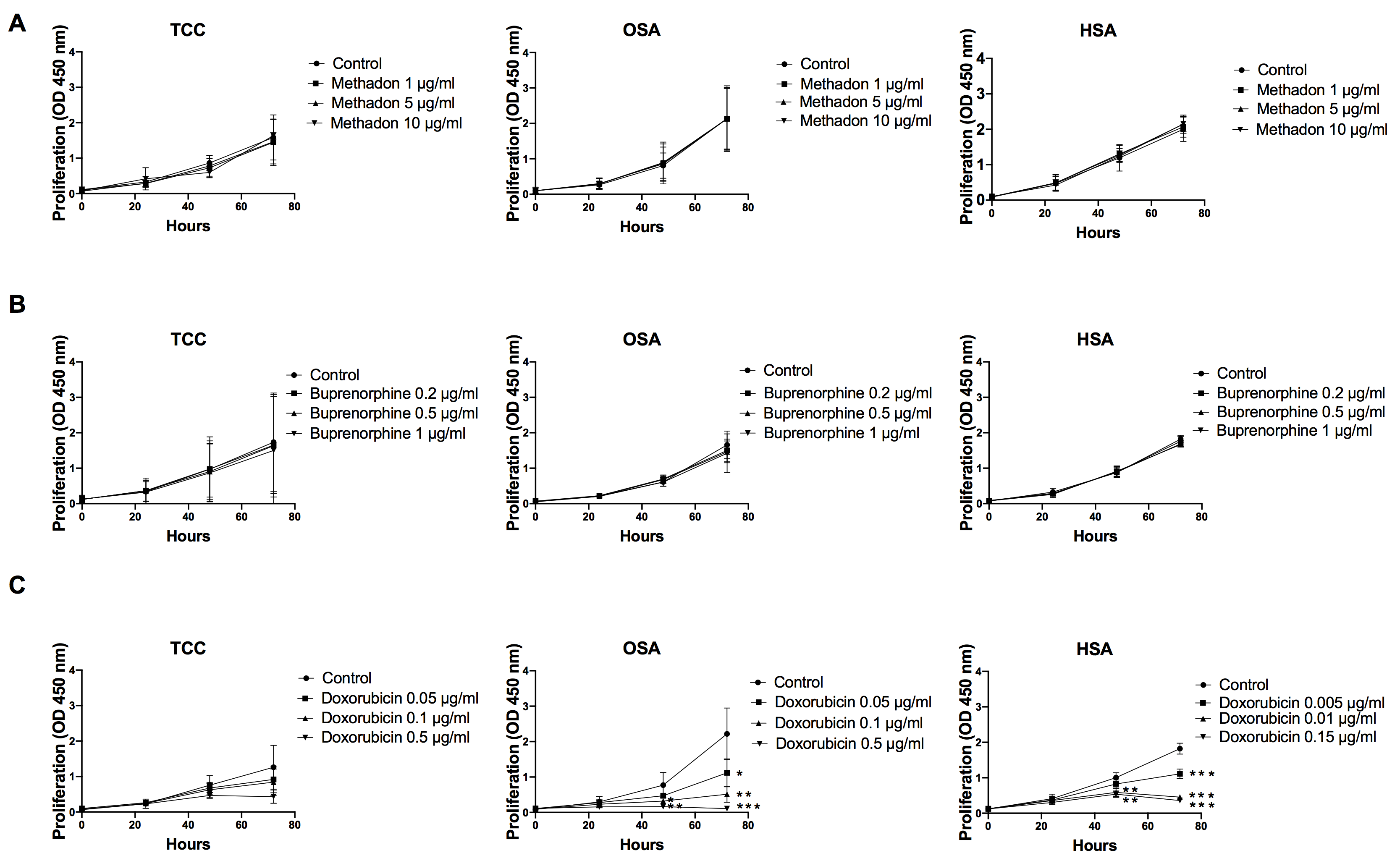

Supplement: Supplementary file 3 — Fig S3 [file VMS3-6-283-s003.tiff]

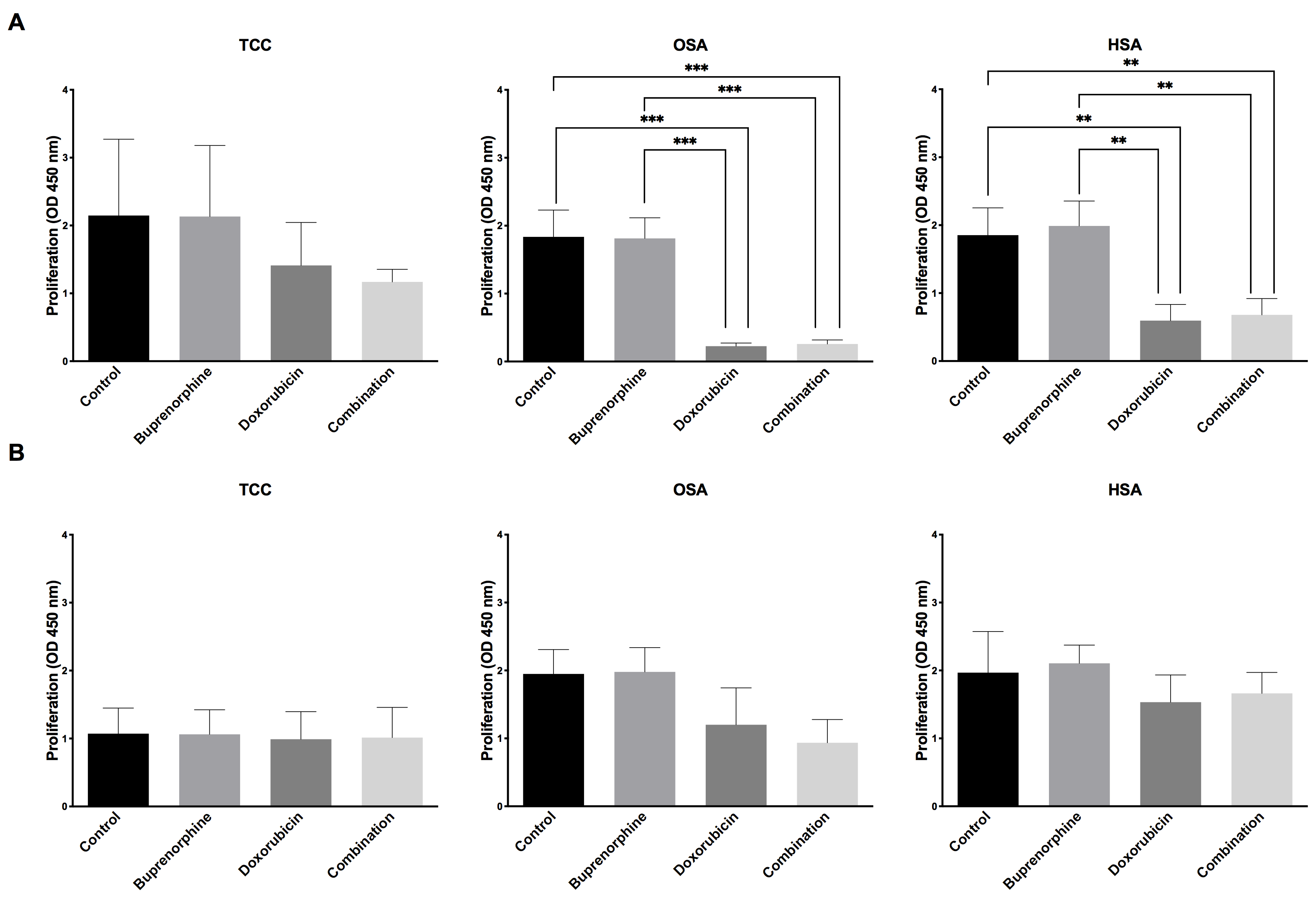

Supplement: Supplementary file 4 — Fig S4 [file VMS3-6-283-s004.tiff]
